# Developing a novel selection method for alcoholic fermentation starters by exploring wine yeast microbiota from Greece

**DOI:** 10.3389/fmicb.2023.1301325

**Published:** 2023-12-20

**Authors:** Aikaterini P. Tzamourani, Vasileios Taliadouros, Ioannis Paraskevopoulos, Maria Dimopoulou

**Affiliations:** ^1^Department of Wine, Vine and Beverage Sciences, School of Food Science, University of West Attica, Athens, Greece; ^2^Department of Statistics and Insurance Science, University of Piraeus, Piraeus, Greece

**Keywords:** Greek terroir, phenotypic diversity, indigenous yeast, wine yeast selection, biostatistics tools

## Abstract

The selection of native yeast for alcoholic fermentation in wine focuses on ensuring the success of the process and promoting the quality of the final product. The purpose of this study was firstly to create a large collection of new yeast isolates and categorize them based on their oenological potential. Additionally, the geographical distribution of the most dominant species, *Saccharomyces cerevisiae*, was further explored. Towards this direction, fourteen spontaneously fermented wines from different regions of Greece were collected for yeast typing. The yeast isolates were subjected in molecular analyses and identification at species level. RAPD (Random Amplified Polymorphic DNA) genomic fingerprinting with the oligo-nucleotide primer M13 was used, combined with Matrix Assisted Laser Desorption Ionization–Time of Flight Mass Spectrometry (MALDI-TOF MS) technique. All yeast isolates were scrutinized for their sensitivity to killer toxin, production of non-desirable metabolites such as acetic acid and H_2_S, β-glucosidase production and resistance to the antimicrobial agent; SO_2_. In parallel, *S. cerevisiae* isolates were typed at strain level by interdelta – PCR genomic fingerprinting. *S. cerevisiae* strains were examined for their fermentative capacity in laboratory scale fermentation on pasteurized grape must. Glucose and fructose consumption was monitored daily and at the final point a free sorting task was conducted to categorize the samples according to their organoleptic profile. According to our results, among the 190 isolates, *S. cerevisiae* was the most dominant species while some less common non-Saccharomyces species such as *Trigonopsis californica, Priceomyces carsonii*, *Zygosaccharomyces bailii, Brettanomyces bruxellensis* and *Pichia manshurica* were identified in minor abundancies. According to phenotypic typing, most isolates were neutral to killer toxin test and exhibited low acetic acid production. Hierarchical Cluster Analysis revealed the presence of four yeast groups based on phenotypic fingerprinting. Strain level typing reported 20 different *S. cerevisiae* strains from which 65% indicated fermentative capacity and led to dry wines. Sensory evaluation results clearly discriminated the produced wines and consequently, the proposed yeast categorization was confirmed. A novel approach that employs biostatistical tools for a rapid screening and classification of indigenous wine yeasts with oenological potential, allowing a more efficient preliminary selection or rejection of isolates is proposed.

## Introduction

The principal metabolic process in wine production is alcoholic fermentation (AF), wherein grape sugars are transformed into ethanol, carbon dioxide through the action of yeast and in parallel a plethora of secondary metabolites are produced (Ribéreau-Gayon et al., 2006; Fleet, 2008; Querol et al., 2018). Although the traditional function of wine yeasts is carrying out alcoholic fermentation, the advent of modern wine microbiology targets to unravel the yeasts properties, in order not only to improve fermentation performance but also wine quality (Barata et al., 2012; Suárez-Lepe and Morata, 2012; Cordero-Bueso et al., 2013; Maicas, 2020).

During fermentation process, the consortium of yeasts is rapidly evolving and shaped by biotic and abiotic factors (Ciani et al., 2004; Jolly et al., 2014; Belda et al., 2016; Sha et al., 2018; Comitini et al., 2021; Dimopoulou et al., 2022). Non-Saccharomyces (NS) yeasts dominate the early stages of fermentation, but the gradual production of ethanol allows the prevalence of the more adaptable species with the most dominant; *Saccharomyces cerevisae* (Lambrechts and Pretorius, 2000; Soden et al., 2000; Clemente-Jimenez et al., 2005; Sadoudi et al., 2012; Liu et al., 2016; Gobert et al., 2017). Other factors besides ethanol, which create a stressful environment, are high sugar concentration (osmotic stress), low pH (acid stress), decreasing oxygen (hypoxia), presence of numerous microorganisms that compete for nutrients or produce inhibitory compounds and also presence of sulfur dioxide (Mateo et al., 2001; Benito et al., 2015; Roudil et al., 2020; Reiter et al., 2021). Nowadays grapes during harvest contain even higher concentrations of sugars due to climate change, rendering the role of yeast even harder and increasing the possibility of stuck or delayed fermentation. Yeast inoculation in wine industry is the key to ensure fermentation flow and sugar depletion (Bely et al., 2008; Benito et al., 2015; Ciani and Comitini, 2015; Dimopoulou et al., 2020). The commercialization of selected autochthonous strains of *S. cerevisiae* to drive alcoholic fermentation is justified by their remarkable adaptability to wine stressors (Fleet, 2008; Rossouw et al., 2012; Reiter et al., 2021). Commercial *S. cerevisiae* strains assure fermentation completion and enhance the standardization and reproducibility of the final product. However, they often lack some unique characteristics linked to biodiversity parameters, and therefore the final wines may lack complexity and typicity (Comitini et al., 2017; Parapouli et al., 2020; Sidari et al., 2021; Christofi et al., 2022).

Targeting the success of alcoholic fermentation and the production of high value wines, producers have focused on the selection of indigenous *S. cerevisiae* strains which have been previously evaluated for their oenological properties to drive AF (Caridi et al., 2002; Le Jeune et al., 2006; Pulcini et al., 2022). Numerous studies focus on the selection of “novel” *S. cerevisiae* with main concern, their improved technological and organoleptic properties; such as high yields of productivity, stress tolerance, unique aromatic characteristics and positive sensory attributes (Capece et al., 2010, 2019; Suárez-Lepe and Morata, 2012; Basa et al., 2022; Tronchoni et al., 2022). Some beneficial oenological traits are alcohol tolerance, lower production of acetic acid and H_2_S, SO_2_ tolerance, neutral killer character and resistance to high concentrations of sugars (de Ullivarri et al., 2011; Comitini et al., 2017; Pulcini et al., 2022). Furthermore, some yeasts possess the enzymes of β-glucosidases, whose activity results in releasing aglycones and this procedure directly affects beneficially the produced aroma (Mansfield et al., 2002). All the abovementioned characteristics are criteria for the selection of starter cultures, resulting in wines with controlled quality and attractive organoleptic profile (Christofi et al., 2022; Pulcini et al., 2022).

The autochthonous yeast strains, which drive and survive until the end of alcoholic fermentation, are usually characterized by high fermentation rate and alcohol tolerance (Suárez-Lepe and Morata, 2012; Gutiérrez et al., 2013; Garofalo et al., 2018). Numerous researchers have previously isolated a large collection of native yeasts and by means of molecular biology, culture-based methods and mini-vinifications have concluded in some strains with oenological perspective (Caridi et al., 2002; Mestre Furlani et al., 2017; Garofalo et al., 2018; Binati et al., 2019). However, this procedure demands time, advanced analysis and special equipment. The rapid elimination of some isolates from a big yeast collection can result in a more practical and cost-efficient way to select new autochthonous strains with oenological interest. The main objective of this research was to classify a large collection of yeast isolates from spontaneously fermented wines produced from various cultivars and regions in Greece, based on their technological properties with oenological interest. A simple and applicable phenotypic-based methodology for rapid preselection of wine autochthonous yeast with oenological potential is proposed. The qualitative data were transformed accordingly and treated by various biostatistical tools in order to achieve a classification method. The proposed HCA on selected phenotypic tests was validated by wine micro-fermentation trials of the 20 isolated *S. cerevisiae* strains and their corresponding sensory attributes.

## Materials and methods

### Origin of the samples

Fourteen samples of spontaneously fermented wines were obtained, from four geographical areas in Greece, namely Goumenissa in northern Greece, Pelion in central Greece, Nemea in southern Peloponnese and the island of Santorini (Table 1). The varieties and the vintage of the wines are noted in Table 1. All samples were collected from dry wines (before SO_2_ addition), with an alcohol level from 12.5% vol to 14% vol. The majority of the wineries have never used commercial *S. cerevisiae* strains to drive alcoholic fermentation, whatsoever for the wineries that do use, the profile of the commercial strains (Supplementary Table S1) has been compared with the isolated strains of the present study.

**Table 1:** Sample coding and geographical origin of the wine samples.

Sample ID	Origin	Variety	Type of wine	Vintage	Isolates
GB	Santorini	Assyrtiko	White	2020	5
A6	Santorini	Assyrtiko	White	2020	15
A26	Pelion	Assyrtiko	White	2020	21
K21	Pelion	Xinomavro	Red	2019	9
K29	Pelion	Xinomavro	Red	2020	18
K23	Pelion	Xinomavro	Red	2020	24
Κ24	Pelion	Xinomavro	Red	2018	21
A30	Nemea	Assyrtiko	White	2020	12
K32	Nemea	Agiorgitiko	Red	2019	18
K33	Nemea	Agiorgitiko	Red	2019	15
K34	Nemea	Agiorgitiko	Red	2019	17
A9	Nemea	Roditis	White	2019	5
A19	Goumenissa	50 Malagouzia/50 Muscat	White	2020	5
K16	Goumenissa	Xinomavro	Red	2020	5

All wines were collected from the wineries at the end of the fermentation process.

### Molecular characterization and identification of microorganisms

#### Colonies isolation and purification

For yeast isolation, 100 μL of wine was directly and aseptically spreaded on WL agar plates (Condalab, Madrid Spain). Plates were incubated at 28°C for 48 h. Each sample was analyzed in duplicate. When there were noted more than 20 colonies by plate, a representative selection of yeast colonies was made from WL plates in accordance with the method described by Harrigan and McCance (1976). Colonies were purified by streaking on YPD agar plates [(g/L): Yeast extract 10, Bacteriological peptone 20, Dextrose (D-Glucose) 20, Agar 20]. Plates were incubated at 28°C for 48 h. Each sample was analyzed in duplicate. Additionally, the cultures were maintained at −20°C in YPD broth supplemented with 30% (v/v) glycerol (Serva, Heidelberg, Germany). Before experimental use each isolate was subcultured twice in YPD broth (at 28°C) for 48 h.

#### Genomic DNA extraction

Total genomic DNA from the yeast isolates was extracted according to the protocol described by Ercolini et al. (2001) modified by adding lyticase at 2.5 U/mL (Lyticase from Arthrobacter luteus, Sigma–Aldrich, Germany) for yeast cell lysis (Bonatsou et al., 2018). Moreover, quantification and quality control of DNA extract was performed by spectrophotometer (Epoch, Biotek, USA) at wavelengths of 260, 280, and 230 nm.

#### PCR fingerprinting

RAPD-PCR analysis was initially used for clustering the isolates, employing the primer M13 (5′-GAGGGTGGCGGTTCT-3′), according to the protocol of Lieckfeldt et al. (1993). PCR amplification was conducted in 20 μL final reaction volumes, containing 5 μL of One Taq Quick-Load Reaction Buffer (New England Biolabs, USA), 1 U of One Taq Quick-Load DNA Polymerase (New England Biolabs, USA), 100 μM of dNTP’s (10 mM), 10 μM of M13 oligonucleotide primer and 20 ng of template DNA. The amplification program consisted of: 30 s of initial denaturation at 94°C, 3 cycles of 30 s at 94°C, 5 min at 35°C, 5 min at 68°C and then 32 cycles of 30 s at 95°C, 2 min at 53°C, 3 min at 68°C, concluding with 3 min at 68°C.

Genetic diversity within *S. cerevisiae* isolates was assessed by interdelta analysis proposed by Legras and Karst (2003) with minor modifications. Briefly, PCR amplifications were carried out in 20 μL reaction containing 2.5 μL of Buffer A 10 X, 0.25 μL of Taq DNA Polymerase (5 U/μL, Kapa Biosystems, USA), 100 μM of each dNTP, 10 μM of each oligonucleotide primer [delta 12 (5′- TCAACAATGGAATCCCAAC-3′) and delta 21 (5′-CATCTTAACACCGTATATGA-3′)]. Amplification reactions were performed with the following conditions: 4 min at 95°C followed by 40 cycles of 30s at 95^°^C, 30 s at 46^°^C and 90s at 72^°^C and a finishing step of 10 min at 72^°^C. In addition to the indigenous *S. cerevisiae* strains, the commercial *S. cerevisiae* strains (Supplementary Table S1) were also examined. The commercial strains were treated as all the other isolates subjected in the fingerprint analysis. All amplifications were carried out in a thermocycler (T100, Biorad, United States).

The products were run on a 1.5% (w/v) agarose gel in 1 × TAE buffer, stained with ethidium bromide (20 min) at 110 V for 140 min and scanned under ultraviolet light (MiniBIS, DNr, Israel). A 100 bp and 1Kb DNA ladder (Nippon Genetics, Germany) served as size standard in RAPD-M13 and interdelta PCR products, respectively. The resulting fingerprints were digitally captured, converted, normalized and analyzed using the Dice coefficient with Bionumerics software version 6.1 (Applied Maths, Sint-Martens-Latem, Belgium). Means of the Unweighted Pair Group Method using the Arithmetic Average (UPGMA) clustering algorithm led to the formation of the species- and strain specific dendrogram. Furthermore, species identification was achieved since two to five representative strains from each different cluster (distance >90%) were selected to species identification by MALDI-TOF MS method as described by Windholtz et al. (2022).

### Screening the technological properties of the isolated yeasts

Important technological characteristics such as production and sensitivity to killer toxin, acetic acid production, β-glucosidase production, resistance to SO_2_ and H_2_S production were tested for the yeast screening (Rodríguez et al., 2004; Comitini et al., 2011; Domizio et al., 2011; Konate et al., 2014). All assays were replicated twice. Precultures were grown in YPD broth at 28°C for 48 h.

#### Production and sensitivity to killer toxin

The killer character determination was performed using the plate assay described by Domizio et al. (2011), with positive activity (K+) recognized by inhibition of growth of the sensitive strain (*S. cerevisiae* SO classic, Martin Vialatte, France), seen as a clear zone surrounding the seeded strain. The *S. cerevisiae* killer strain VIN13, (Anchor, France), showing killer activity, was used as positive control. Sensitive character was observed when colonies could not grow onto agar substrate which was poured with a killer yeast strain (*S. cerevisiae* VIN13, Anchor, France); the isolate was designated as sensitive (K-, R-). The *S. cerevisiae* sensitive strain (SO classic, Martin Vialatte, France), was used as positive control. Yeasts with negative reaction to the killer character (K-) and negative sensitivity (R+) were characterized as neutral (de Ullivarri et al., 2011). The *S. cerevisiae* killer strain VIN13, (Anchor, France), showing killer activity, was used as positive control.

#### Acetic acid production

Acetic acid production *was* noticed by formation of clear zones around colonies of the strains which were implemented and spotted on Hestrin-Schramm CaCO_3_ agar (Aydin, 2009). This medium was composed of [g/L: CaCO_3_ 5.0, Yeast extract 3.0, Agar 15.0 and Dextrose 15.0] (Konate et al. 2014). Cultures were incubated at 28°C for 5 days. The ability of the colonies to form clear zones through the hydrolysis of the white salt was considered as positive reaction to this test.

#### β-glucosidase production

The β-glucosidase activity was evaluated as described by Rodríguez et al. (2004) on agar plates containing arbutin as substrate. Screening was carried out on agar plates with arbutin as substrate [g/L: Yeast Nitrogen Base/YNB (Condalab, Madrid Spain) 6.7, arbutin (Sigma Aldrich, USA), 5, agar, 20]. The pH was adjusted to 5.0 and after sterilization 2 mL of a sterile 1% (w/v) ferric ammonium citrate solution was added to 100 mL of melted medium. Each plate was inoculated by spot assay, incubated at 28°C and examined after 8 and 15 days. Enzymatic activity was noticed visually when brown color develops in the agar.

#### Resistance to SO_2_

The SO_2_ resistance was determined by screening on plates with synthetic substrate. Based on the protocol described in detail by Comitini et al. (2011), the isolates were inoculated onto YPD agar plates at pH 3 (with citrate–phosphate buffer), added with increasing doses of K_2_S_2_O_5_ in different concentration corresponding to 0, 100, 200, 300, 400, 500 mg/L of free SO_2_ and incubated at 28°C. The yeast growth was observed after 2, 5 and 8 days after the inoculation.

#### H_2_S production

H_2_S potential production was estimated by spreading the yeasts onto Biggy Agar (Condalab, Madrid, Spain) (Domizio et al., 2011). On this medium, H_2_S-positive isolates create brown colonies, while H_2_S negative isolates create white colonies. The H_2_S effect was noticed after 2, 5 and 8 days of incubation at 28°C when the color of the colonies was recorded. The following arbitrary scale was used: 0 = white (no production); 1 = light brown; 2 = brown; 3 = dark brown.

### Screening on grape must (micro-fermentations)

The fermentation potential of the yeast strains was evaluated in micro-fermentation trials. Fermentations were carried out at 18^°^C in 50 mL of pasteurized (72^°^C, 10 min) Assyrtiko must which was provided by Gaia (Sanotrini, Greece) winery (vintage 2021) under static conditions. The initial pasteurized grape must (pH = 3.2, total acidity = 5.77 g tartaric acid/L, YAN = 609 mgN_2_/L, 16.6 mg SO_2_, 5.1 free SO_2_) contained 119.5 g/L glucose and 120.1 g/L fructose. Precultures were grown in YPD broth at 28^°^C for 48 h, and then used to inoculate each fermentation (10^6^ cell/mL). Residual sugar (glucose and fructose) determination was performed on the wines in daily basis using Enzytec kit-liquid Glucose-Fructose (r-biopharm, Germany). All fermentations were assessed duplicated and analysis was conducted twice.

### Free sorting task

In the end of the fermentation all produced wines were categorized based on their aromatic profile (odor) by means of a free sorting task test (Sáenz-Navajas et al., 2012). A total of 10 experienced panelists participated in the pilot study. Participants were provided with the produced dry wine samples (15 mL) in ISO approved wine glasses coded with different three-digit numbers and arranged in random order. Participants were asked to sort the 13 wines on the basis of similarity attending to the global sensations perceived in nose (p.e. intensity, floral, fruity, off odor characteristics). Panelists could make as many groups as they wished. Upon completion, they recorded the three-digit codes of the samples of each group on a paper sheet. All wines were served at room temperature. The sessions took place in a ventilated and air-conditioned tasting room (at around 20^°^C). Panelists were not informed about the nature of the samples.

### Data analysis

#### Univariate analysis

Data obtained from the phenotypic tests, were converted into numerical from character data (+, −) as it is illustrated in Table 2. Consequently, data are further investigated by means of statistical analysis. Firstly, MANOVA was attempted but the scaled data of H_2_S experiment during the MANOVA application process could not satisfy its application assumptions. MANOVA assumes multivariate normality and homogeneity of variance–covariance tables between groups. These assumptions were not met, and we considered a univariate analysis using ANOVA more appropriate for each variable. Univariate method may offer simpler and more straightforward interpretations of the results. Using ANOVA for each dependent variable separately allows focusing on each variable’s unique response to the independent variables. More specifically, the possibility of having statistically important differences was examined at origin and species level. Therefore we apply the model Yij = μi + εij, i = 1, 2, 3, …where with i we denote the levels of the H_2_S factor and j = 1, 2, … the observations we have for each level (Koutras and Evagelaras, 2010). In the current analysis the average level of H_2_S production per origin or species was considered as a dependent variable Y and time of incubation as variable X. To obtain safe statistical conclusions, (a) the assumption of equality of dispersions at the levels of factor at the level of significance of 5% and (b) the test of the normality and independence of errors at the level of significance of 5% were carried out. To check the equality of variations, Levene’s test was used, where we do not reject the zero hypothesis to be checked and therefore ensure homoscedasedality. Then, to check the normality and independence of the errors, Studentized residuals were used. Utilizing the non-parametric test of Kolmogorov–Smirnov does not reject the null hypothesis that errors follow Normal Distribution. Regarding the test of independence of errors, the non-parametric, Run’s test was used and the null hypothesis that errors are independent cannot be rejected. Having ensured the above conditions, the test for whether there are significant differences between the levels of the factor is of the form:


$${Η}_{0}:\mu_{1}=\mu_{2}=\mu_{3}vs\;{Η}_{1}:\mu i\neq \mu j\;for\;a\;\mathrm{combination}\;\left(i,j\right),i\neq j$$

The value of *p* of the test is less than 0.05 and therefore at a significance level of 5%, zero assumption that there are no significant differences in the levels of the factor is rejected.

**Table 2:** Phenotype coding based on the character that resulted after the five different tests.

Digit	Test	Characterization
1st	Killer	1 = neutral, 2 = sensitive
2nd	H_2_S	0 = no production, 1 = low production, 2 = high production, 3 = very high production
3rd	Acetic acid	0 = production, 1 = no production
4th	SO_2_ resistance	0 = no resistance, 1 = resistance until 300 μg/L, 2 = resistance between 400–500 μg/mL
5th	β-glucosidase	0 = positive response, 1 = negative response

#### Classification

Hiercharchical Cluster Analysis (HCA) of the different phenotypes based on the results of the five phenotypic tests was performed under R (3.6.2) software using Euclidean distance and Ward method. Among various hierarchical clustering methods such as Single Linkage Method, Weighted Average Linkage Method, Centroid Method, Flexible Strategy Method etc., the Ward’s Method was chosen as it is the most effective. This method differs from others and is designed to minimize variance within groups. In particular, the method has some very good properties and usually creates groups with a similar number of observations. The development of logical rules that lead to finding the optimal number of groups of a dataset has occupied several researchers active in the field of cluster analysis, since it is obvious that this problem is of great practical interest. Thorndike (1953) proposed a graphical approach to the problem whereby an axis is first depicted on one axis of the average within-cluster distance and on a second axis of the number of groups. With each increase in the number k of groups there is a corresponding decrease in the average distances within the groups. In most cases a position appears where we have a sharp decrease in the average distances within the groups and then “leveling” the graph. In order to find the number of groups the datagram resulting from a hierarchical cumulative method was examined and from it determine the optimal number. More specifically, at that point of the dendrogram where the greatest change in the quantity recorded on the horizontal axis (distance) is observed, we can bring a parallel line to the vertical axis and see at how many points the datagram intersects. The number k for which we observe large concatenation distances relative to the previous one (k-1 groups) is a reasonable value for the optimal number of groups. For this reason, 4 groups were selected.

**Figure 1:**
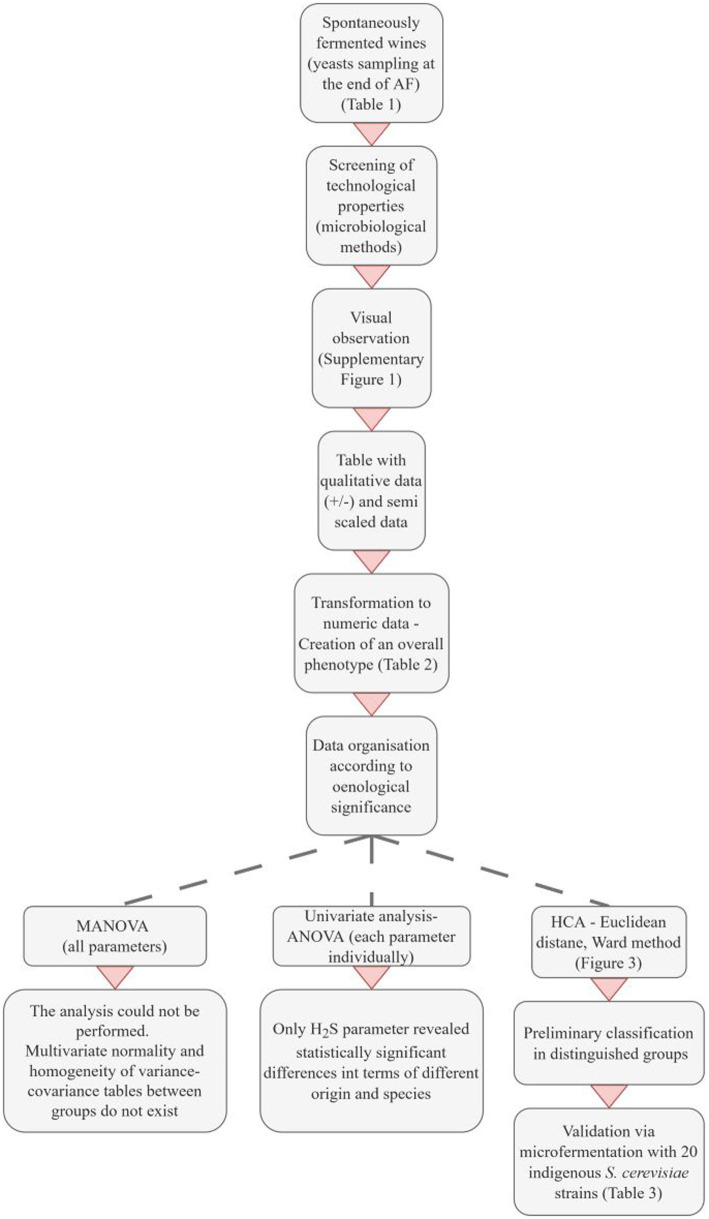
Overview of the development of the proposed phenotypic classification method. AF, Alcoholic fermentation; HCA, Hierarchical cluster analysis.

Based on the phenotypic test results, we encoded the positive and negative responses to the microbiological phenotypic assays. The data were organized according to oenological significance to establish an overall phenotype. Priority was given to the production of killer toxin, followed by H_2_S production, acetic acid production, SO_2_ resistance, and, finally, β-glycosidase activity. All parameters were considered, with particular attention to the sequence of data. This arrangement was determined with oenological requirements in mind, aiming for yeast strains that are insensitive to killer toxin, are low producers of H_2_S or acetic acid, exhibit resistance to SO_2_, and possess desirable β-glucosidase activity. Α detailed description is provided of the development of the proposed selection method (Figure 1).

#### Sensory analysis

Encoding free sorting data was the key to categorize wine samples based on the results of the sensory assessment. For each group, results are encoded in an individual similarity matrix (wines × wines), in which 1 stand for two wines set in the same group and 0 for two wines put in different groups. These individual matrices are summed across subjects; the resulting co-occurrence matrix represents the global similarity matrix where larger numbers indicate higher similarity between samples. The assumption underlying this method is that samples grouped together are more similar than samples sorted into different groups. The resulting cooccurrence matrix was submitted to HCA (Ward method) in order to derive a spatial arrangement of wines with R (3.6.2) software analysis.

## Results

**Figure 2:**
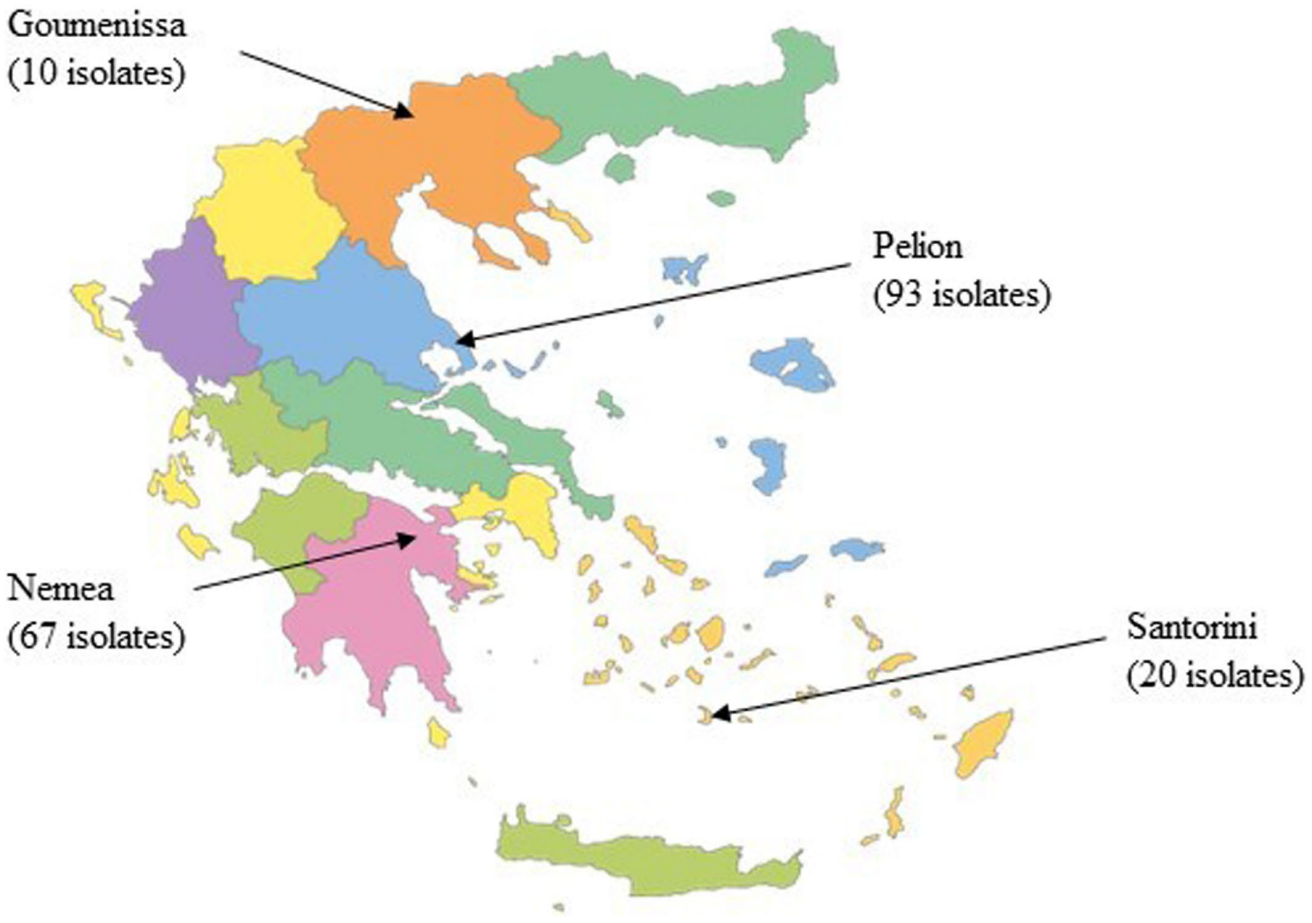
Geographical distribution of the yeast isolates.

The community structure of yeast in wine samples collected directly from wineries was determined at the end of alcoholic fermentation (AF). A total of 14 wine samples were collected, including two from Santorini, five from Pelion in central Greece, five from Nemea in southern Peloponnese, and two from Goumenissa in northern Greece (Table 1). A total of 190 yeast isolates were obtained, and their geographic origins are shown in Figure 2. The obtained RAPD-M13 PCR fingerprints were clustered using UPGMA analysis with Dice as a coefficient, and a representative number of isolates from each cluster were identified using MALDI-TOF MS. Six different species were identified, namely *Saccharomyces cerevisiae* (168 isolates), T*rigonopsis californica* (1 isolate), *Brettanomyces bruxellensis* (5 isolates), *Zygosaccharomyces bailii* (8 isolates), *Priceomyces carsonii* (1 isolate), and *Pichia manshurica* (7 isolates). Specifically, *S. cerevisiae* was the most dominant species with the isolation frequency exceeding 88.4% (data not shown). Although 7 isolates of *P. manshurica* were found, all of them were obtained from a single sample and there was no repetition across samples. Thus, it is not possible to make assumptions based on the presence of a random spoilage yeast/fungus species in just one sample. Conversely, *Z. bailii* was detected in minor amounts in 5 different samples from 3 different regions (Nemea, Goumenissa, and Pelion).

### Technological properties of the isolates

All 190 isolates were subjected to some phenotypic tests to monitor their technological properties. These yeasts were analyzed for different characteristics, such as killer activities, acetic acid production, β-glucosidase activity, hydrogen sulphide production and sulphur dioxide resistance, revealing both inter-generic and intra-generic biodiversity.

Among our observations, only 12% of the yeasts (23 isolates) were characterized as sensitive to killer toxin and the rest were noted as neutral. No killer yeast was reported. Among these 23 yeasts the only isolate of *T. californica* is sensitive to killer toxin, 20 isolates belong to *S. cerevisiae* and 2 isolates of *Z. bailii*. 137 yeasts were observed to produce clearly acetic acid and thus forming clear zones around the colony in CaCO_3_ agar; whereas 53 did not produce acetic acid. From these isolates, *T. californica* made the only exception since the rest 52 isolates belonged to *S. cerevisiae* group. All yeasts showed low or absence of β-glucosidase activity because no color change from white to brown/dark brown was noticed. However, 12 colonies (8 *S. cerevisiae*, 1 *T. californica* and 3 *Z. bailii*) were slightly darker, compared to the others, revealing low enzymatic activity (6,3% of the total isolates). Moreover, regarding the potential H_2_S production at species level *B. bruxellensis* followed by *T. californica* and *P. carsonii* perceived significantly the highest levels of H_2_S. All isolates of *Z. bailii* proved to be low H_2_S producers while *S. cerevisiae* isolates expressed great variability. Sulphur dioxide resistance, a very desirable oenological characteristic, was determined to be a common trait to almost all tested isolates. Only 6, 12 and 11 isolates were sensitive at the concentrations of 100 mg/L, 200 and 300 mg/L, respectively. It is noteworthy that SO_2_ inhibited the growth of most *Z. bailii* isolates and 82% of the isolates were resistant to the extreme concentrations of 400 and 500 mg/L.

Hydrogen sulphide production was the only phenotypic characteristic which exhibited statistically significant differences in terms of origin and species characterization. The production of H_2_S was measured at 2, 5 and 8 days and color grading indicated its quantity [0 = white (no production); 1 = light brown; 2 = brown; 3 = dark brown]. In the current analysis the average level of H_2_S production (0,1,2,3) was considered as a dependent variable Y per origin and species (data not shown), and time of incubation as variable X that takes the values 1: for H_2_S that was produced after 2 days of incubation, 2: for H_2_S that was produced after 5 days of incubation, 3: for H_2_S that was produced after 8 days of incubation. Test of homogenicity of variances, one sample Kolmogorov–Smirnov test and test off between-subjects effects proved that significant differences between the levels of factor H_2_S-production exist in terms of origin and species. Isolates from the regions of Nemea and Pelion revealed lower levels of H_2_S production, regardless of the species parameter. Moreover, *B. bruxellensis* followed by *T. californica* and *P. carsonii* perceived significantly the highest levels of H_2_S. More specifically, *B. bruxellensis* released more H_2_S at 5th and 8th day, whereas *T. californica* and *P. carsonii* emissions did not change after the 2nd day. *Z. bailii* proved to be low H_2_S producers, *P. manshurica* fair producer and *S. cerevisiae* strain-dependent.

### Preliminary categorization of the isolates

**Figure 3:**
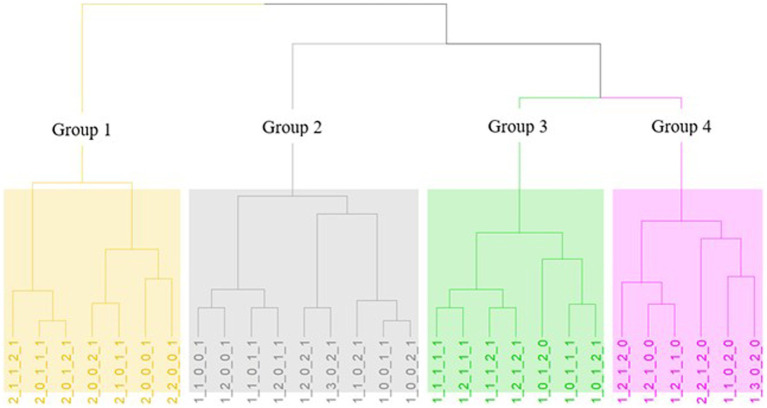
Hierarchical cluster analysis (HCA) of the different phenotypes with Eudlidean distance and Ward method. The four groups are distinct by colors: Group 1 = yellow, Group 2 = grey, Group 3 = green and Group 4 = pink.

**Figure 4:**
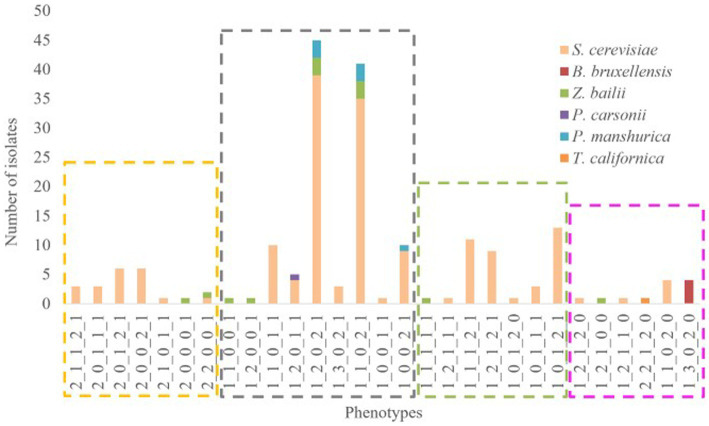
Composition plot of species among the different phenotypes. The captions distinguish the groups which were obtained from HCA (Group 1 = yellow, Group 2 = grey, Group 3 = green and Group 4 = pink).

A novel approach that employs biostatistical tools for rapid screening and classification of large collection of indigenous wine yeasts, allowing for efficient isolate selection is introduced. Following the results of the screening tests, the positive or negative responses to the phenotypic assays were coded (Table 2) and an overall phenotype has been created. In total, 29 different phenotypes were observed. Hierarchical cluster analysis was performed and four main clusters/groups were finally obtained (Figure 3). The algorithm could discriminate the phenotypes based on the 5 tested different parameters. The first group (yellow) consists of isolates which were characterized as sensitive to killer toxin and consequently the yeasts of this group cannot be proposed as starter cultures. Additionally, the second group (grey) is characterized by neutral, no acetic acid production, low resistance to SO_2_, no β-glucosidase activity and varies regarding H_2_S production. The third group (green) includes neutral yeasts, with low H_2_S production, positive acetic acid productivity and absence of β-glucosidase. Finally, in the last group (pink) belong neutral to Killer yeasts (with one exception), high production of H_2_S and acetic acid but resistant to SO_2_ and possible β-glucosidase activity. Therefore, the most preferred groups are the 2nd – grey and the 3rd – green. Additionally, species allocation among phenotypes was also examined (Figure 4). The isolates of *S. cerevisiae* and *Z. bailli* were distributed in the four created phenotypic groups. On the contrary *P. manshurica* and *B. bruxellensis* only in the second and fourth group, respectively.

### Strain identification of *Saccharomyces cerevisiae* isolates

**Figure 5:**
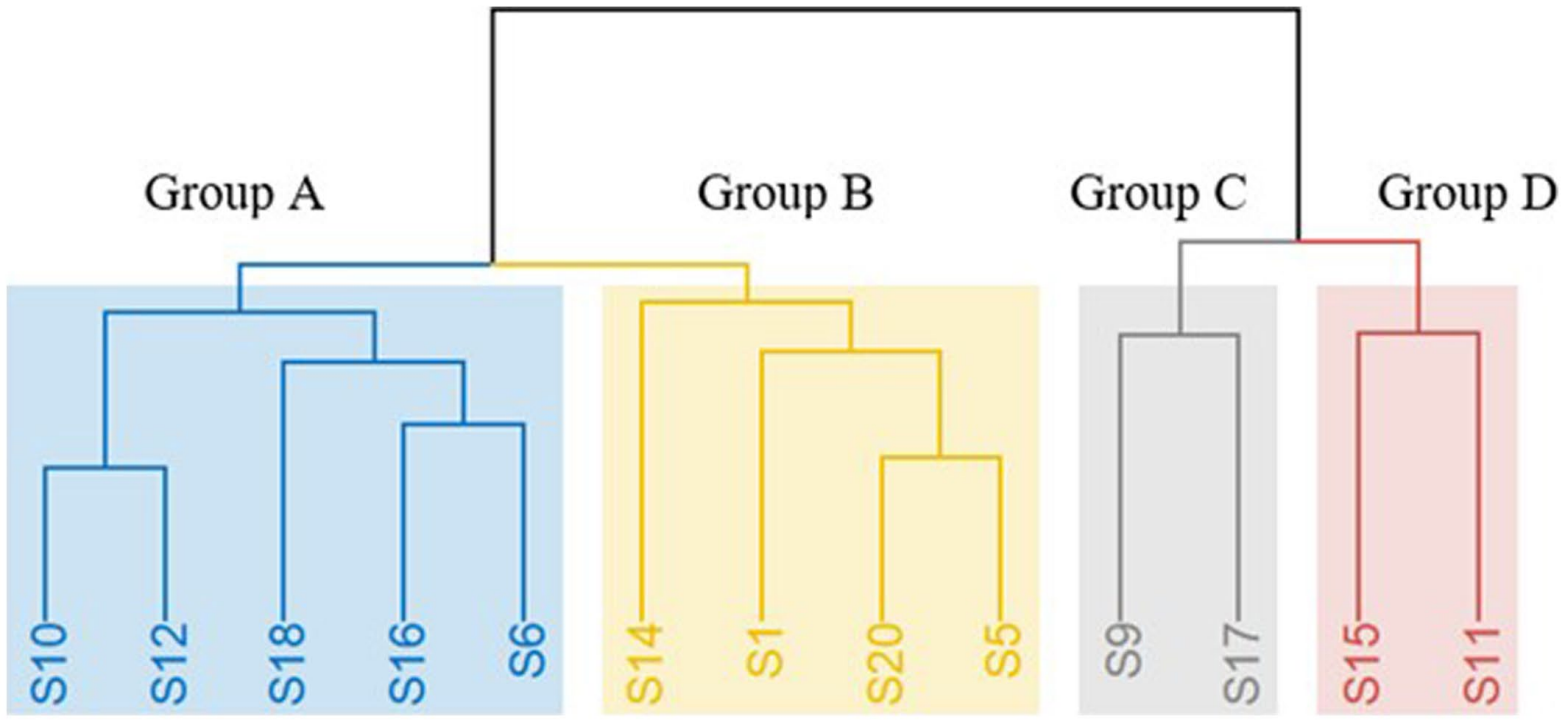
Hierarchical cluster analysis (HCA) of the different aromatic profiles of the 13 produced dry wines from different *S. cerevisiae* strains, based on the results of the free sorting task with Ward method. The four groups are distinct by colors: Group A = yellow, Group B = grey, Group C = green and Group D = pink.

**Figure 6:**
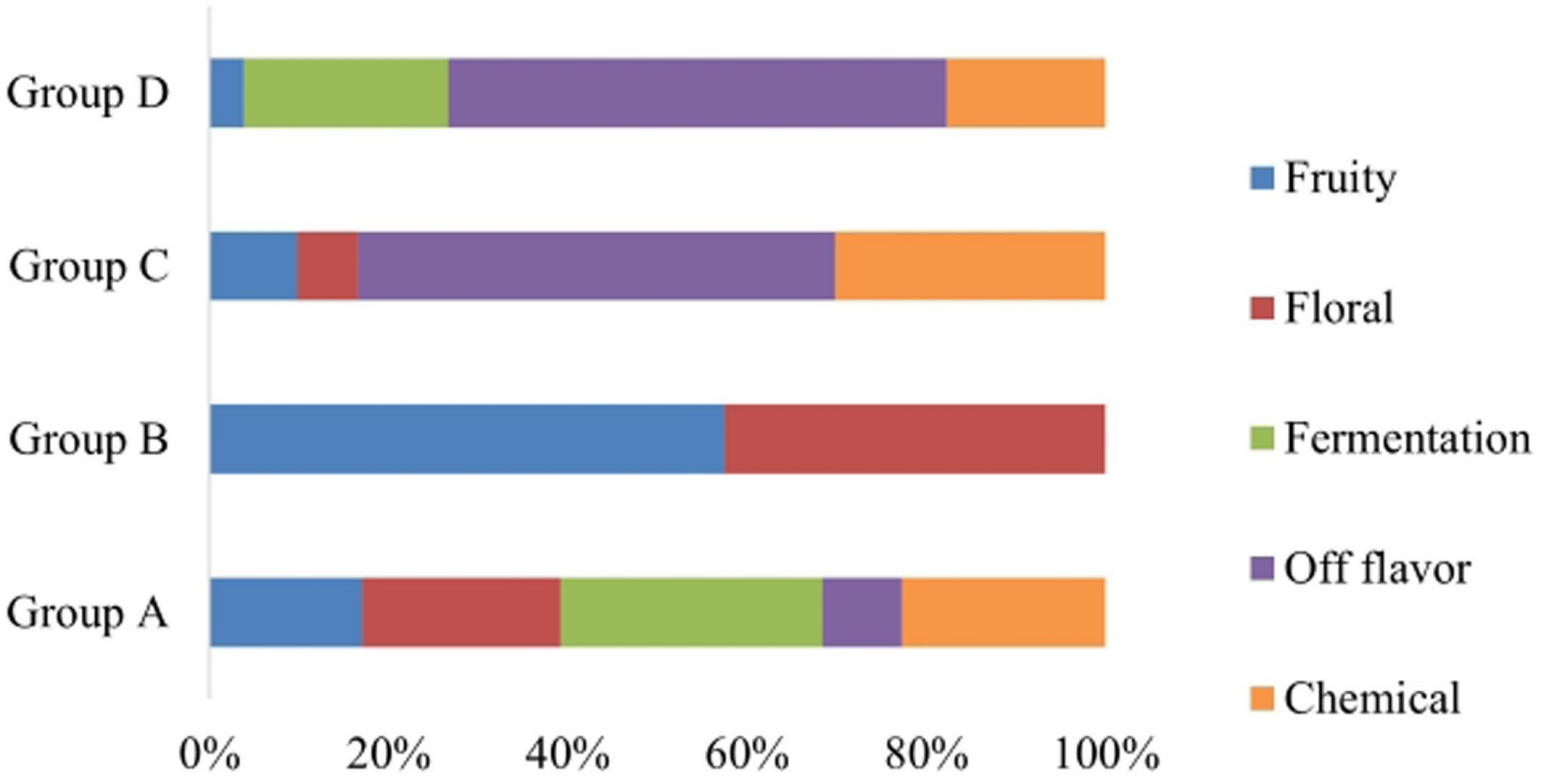
% Composition plot of the descriptors defining each cluster (Group A, Group B, Group C, and Group D) according to the panelists of the sensory assessment.

During spontaneous fermentation process, non-Saccharomyces yeasts dominate at the beginning of AF and the conversion of sugars into ethanol is completed by *S. cerevisiae* yeasts (Di Maro et al., 2007; Garofalo et al., 2016). Consequently, validation of the proposed categorization and also the interest for additional insights into the geographical distribution was focused on the isolates which were identified as *S. cerevisiae*. Strain typing of *S. cerevisiae* revealed the existence of 20 distinct strains performing interdelta PCR, namely S1 to S20. To assess the performance of each *S. cerevisiae* strain under fermentative conditions, laboratory-scale fermentations were conducted, and sugar consumption was measured on a daily basis. It was observed that only 35% of the inoculated *S. cerevisiae* strains (S2, S3, S4, S7, S8, S13, S19) had a lower ability to catabolize sugars, resulting in wines with residual sugar levels of greater than 10 g/L. Therefore, those strains are not suggested to be used as starter cultures. Based on the sensory results of free sorting task, four main clusters were identified (Figure 5). The wine samples that were clustered in groups C and D based on Hierarchical Cluster Analysis (HCA) were deemed as undesirable wines (S). On the other hand, Group B exhibited floral and fruity characteristics, while Group A presented a more complex aromatic profile, which was highly preferable (Figure 6). Hence, the proposed strains are those belonging to groups A and B and it is noteworthy that S1, S10, S14, and S20 produced the most desirable wines with no problematic catabolism of sugars and different organoleptic perception.

Table 3 clarifies that the current findings are in line with the preliminary selection of yeasts, as all isolates from the same strain were also grouped in the same cluster based on their phenotypic characteristics. More specifically, from the preliminary selection, the green cluster (Group 3) and the grey cluster (Group 2) consisted mostly of strains from Group A and B, along with some strains that could not catabolize all sugars, namely S1, S7, S10, S13, S18, and S20 for the green cluster, and S5, S6, S8, S12, S14, S16, and S19 for the grey cluster. Additionally, strains that resulted in abnormal fermentations with off-odor characteristics were clustered in the less beneficial groups: 5 strains in the yellow/Group 1 (S2, S3, S4, S9, and S17) and two in the pink/Group 4 (S11 and S15).

**Table 3:** Fermentation capacity, sensory profile, preliminary group classification, and validation of the classification of the 20 different *S. cerevisiae* strains.

Strain	Fermentation capacity	Sensory profile	Preliminary group classification	Validation of the classification
S1	**✓**	Group B	3-Green	**✓**
S2	X	-	1- Yellow	**✓**
S3	X	-	1-Yellow	**✓**
S4	X	-	1-Yellow	**✓**
S5	**✓**	Group B	2-Grey	**✓**
S6	**✓**	Group A	2-Grey	**✓**
S7	X	-	3-Green	**✓**
S8	X	-	2-Grey	**✓**
S9	**✓**	Group C	1-Yellow	**✓**
S10	**✓**	Group A	3-Green	**✓**
S11	**✓**	Group D	4-Pink	**✓**
S12	**✓**	Group A	2-Grey	**✓**
S13	X	-	3-Green	**✓**
S14	**✓**	Group B	2-Grey	**✓**
S15	**✓**	Group D	4-Pink	**✓**
S16	**✓**	Group A	2-Grey	**✓**
S17	**✓**	Group C	1-Yellow	**✓**
S18	**✓**	Group A	3-Green	**✓**
S19	X	-	2-Grey	**✓**
S20	**✓**	Group B	3-Green	**✓**

**Figure 7:**
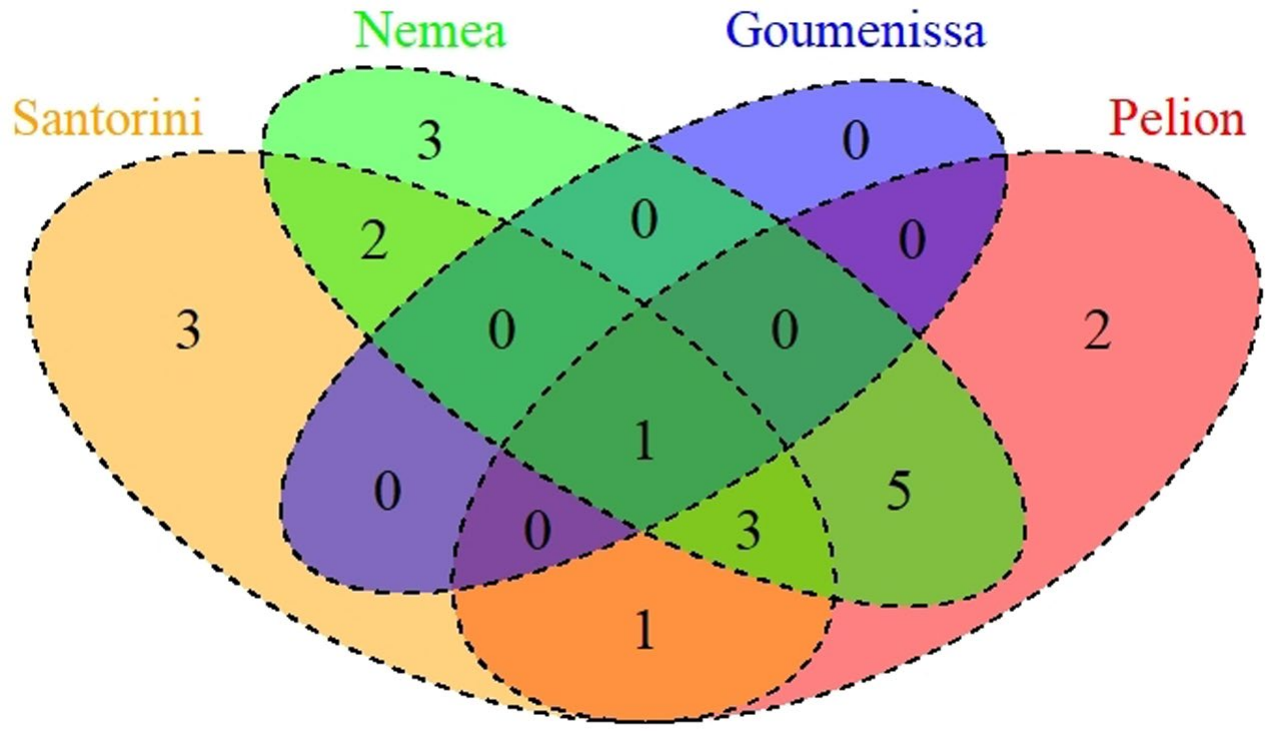
Venn diagram showing the number of unique and shared yeast species per sampling region.

Finally, the geographical distribution of the isolated *S. cerevisiae* strains was examined. Three distinct types of *S. cerevisiae* communities were identified among the investigated samples. The first type consisted of wine samples (GB, A9, K16, A19) with up to two different strains, indicating the prevalence of only one or two strains. The second type consisted of samples that were observed with three to four different strains, while the third type included samples A6, K29, K32, and K33, which exhibited the most complex *S. cerevisiae* community structure. In these samples, seven to eight different strains interacted and survived until the end of the alcoholic fermentation process. Additionally, how the contribution of geographical origin, type of wine, and vintage effects to the dispersion of *S. cerevisiae* strains was investigated. Venn diagrams are illustrated in Figure 7, revealing that one unique strain (S10) was isolated from all tested regions, while three strains were found only in Santorini (S15, S17, S18), three only in Nemea (S11, S12, S13) and two Pelion region (S1, S2). Based on the current results, 10 strains (S2, S3, S4, S5, S6, S7, S8, S9, S10, S20) were found in both red and white wines, while 6 strains (S1, S11, S12, S13, S14 and S19) and 4 strains (S15, S16, S17 and S18) were isolated only from red and white wines, respectively.

## Discussion

Nowadays climate change leads to even more stressful conditions for wine yeast, due to the higher concentrations of sugars on grapes during harvest. Consequently, commercial yeasts, which have been isolated in the past, may not be able to adapt to this new challenging environment (Fleet, 2008; Rossouw et al., 2012; Reiter et al., 2021). The commercialization of new selected indigenous strains to drive alcoholic fermentation is a necessity for the wine industry. Therefore, time efficient methods are crucial when isolating new strains. The process of screening and selecting wine yeasts typically involves several sequential steps (Sidari et al., 2021; Pulcini et al., 2022). However, it is necessary to initially exclude certain isolates when dealing with a vast collection of yeast isolates during the selection process. Assessing technological properties through phenotypic plating methods is a well-established approach. However, manual selection and rejection may not always be feasible especially when no ideal combination of technological properties is discernible, as suggested in previous studies (Mestre Furlani et al., 2017; Sidari et al., 2021).

Yeast selection is a very interesting field not only in wine microbiology, but also in food microbiology. Previous research on indigenous strain selection focused on examining similar characteristics (Caridi et al., 2002; Settanni et al., 2012; Aponte and Blaiotta, 2016). Numerous researchers promote the isolation and selection of indigenous microbiota from various fermented products such as table olives and cheese in order to promote the quality of the final product (del Castillo et al., 2007; Bleve et al., 2015; Bonatsou et al., 2015). In the present study, a similar selection process for indigenous strains was followed, but additionally all the phenotypic results were coded analyzed and further categorized. To the best of our knowledge, no other relevant work managed to transform the qualitative data into numeric in order to perform HCA. Up to now phenotypic results are mostly depicted by tables with negative and positive response and the selection was resulting manually. The proposed idea is to find a way to allow the preliminary rejection of some isolates among a plethora of isolates where no perfect or worst combination is noticed. Qualitative data were transformed into numerical values, and the phenotypic characteristics were arranged based on their impact on the predominance of the selected strain and on the quality of the final product. All isolates were obtained from wines that reflect tolerance to high alcohol and sugar concentrations. Even though no perfect phenotypic combination was noticed, this classification allows the categorization of a large collection of isolates under more than one parameter in a more efficient, low-cost and rapid way.

One necessary trait for a strain, in order to be used as a starter culture, is tolerance to killer toxin (Liu et al., 2015). In the current study, the first digit reveals the killer character and the majority of the isolates were classified as neutral. Previous research has also indicated that autochthonous yeasts are predominantly sensitive or neutral (Comitini et al., 2011; Domizio et al., 2011; de Ullivarri et al., 2014; Velázquez et al., 2016). The lack of killer properties in the isolated yeasts of the current survey, justifies the strain biodiversity among wine samples at the end of AF (Puyo et al., 2023). Furthermore, a brief overview of the literature over the past few decades supports that the most preferable strains should be characterized by non or low production of H_2_S in order to be used as starter cultures (Caridi et al., 2002; Settanni et al., 2012; Aponte and Blaiotta, 2016). Thus, H_2_S production is evaluated as the second most important oenological characteristic of those examined. From another point of view, organic winemaking process emphasizes on the higher risks of oxidation, microbial contamination and H_2_S production. Hence, new starters ‘low H_2_S – SO_2_ – acetaldehyde producers’ are desired (Comitini et al., 2017). Additionally, acetic acid screening was performed because some non-Saccharomyces yeasts produce undesirable concentrations of acetic acid and ethyl acetate from sugars, regarded as unsuitable for winemaking (Caridi et al., 2002; Rodríguez et al., 2004). Moreover, recently *S. cerevisiae* and *Z. bailii* adaptive response and tolerance to acetic acid have been investigated based on functional and comparative genomics strategies (Palma et al., 2018; Capece et al., 2022). Sulphur dioxide resistance is a very desirable oenological characteristic and most studies have been conducted in autochthonous *S. cerevisiae* strains, showing high resistance of this species to SO_2_ and differentiation at strain level (Divol et al., 2012; Settanni et al., 2012). Although legacy allows up to 150–200 mg/L SO_2_ addition for dry wines, while in exceptional cases it can reach up to 400 mg/L for some sweet wines, in the present study the isolates were tested in more extreme concentrations such as 500 mg/L of free SO_2_ (OIV, 2023). Consequently, higher concentrations of SO_2_ can delay the growth of these isolates, but we have to take into account that the experiment is designed *in vitro* and intermediate concentrations were not examined. Yeasts are the main producers of β-glucosidase which is an important enzyme for the hydrolysis of grape glycosides during winemaking. The importance of glycoside hydrolysis in aroma, flavor, color, and color stability was underlined previously (Mansfield et al., 2002; Settanni et al., 2012; Zhang et al., 2021). Modern winemaking techniques often use specific strains of Saccharomyces or non-Saccharomyces yeasts with known β-glucosidase activity to compensate the insufficient enzyme activity in grapes (Aponte and Blaiotta, 2016). Almost all of the abovementioned results are in line with previous studies, in which it is stated that β-glucosidase activity of the species *S. cerevisiae, P. carsonii* and *Z. bailii* is mostly low or even absent (Rodríguez et al., 2004; Cordero-Bueso et al., 2013; Aponte and Blaiotta, 2016; Vilela, 2020; Zhang et al., 2021). On the contrary, our results do not align with those of previous investigations regarding *P. manshurica*, where many species in the Pichia genus have been characterized for their moderate to high β-glucosidase producing ability and the enhance of beneficial volatile compounds in the final product (Zhang et al., 2021; Perpetuini et al., 2020).

The isolates’ classification was validated by the species and strain dispersion. After pilot fermentations assay was conducted, the majority of *S. cerevisiae* strains completed successfully AF and led to wines with exceptional sensory characteristics. Based on recent literature, it is also mentioned that *B. bruxellensis,* in general, intensifies the off-flavor characteristic by producing high concentrations of H_2_S (Avramova et al., 2018). According to the results presented in this study, *B. bruxellensis* isolates were all clustered in the group with the highest H_2_S production. Notably, there is significant intra-species variability, particularly between *S. cerevisiae* and *Z. bailii* species. The validation of the proposed categorization is further validated by the fact that all isolates belonging to the same strain of *S. cerevisiae* exhibited the same phenotypes without exceptions. It is important to highlight that the strains with the most preferable sensorial characteristics were clustered in the 2nd and 3rd group and the opposite. The evaluation of the alcoholic fermentation and produced wines was achieved by monitoring the sugar consumption and the basic sensory evaluation of the final product. The aim was to be able to discriminate the wines based on their basic organoleptic characteristics, that are also examined by the clustering method [pe off odor aromas (acetic acid, H_2_S), fruity/floral aromas (β- glucosidase)], in order to examine the correctness of the proposed classification. The kinetics of alcoholic fermentation reveals the possibility of having a stacked or delayed fermentation. A comparative sensory analysis was chosen in order to evaluate the final product in a more global point of view. According to previous research the free sorting task is an efficient technique for assessing the perception of a set of products by a panel of subjects (Courcoux et al., 2015). This holistic and non-verbal task is an effective tool to be used in product development. Moreover, is a technique that is widespread also in the wine science (Rossouw and Bauer, 2016; Binati et al., 2020).

Hence, the proposed coding and classification method offers the advantage of not requiring expensive molecular techniques and provides valid results within a short timeframe of only two days. Biostatistical tools enhance the categorization of a large collection of yeasts based on their phenotype and allow a preliminary selection of the isolates. This preliminary rejection is time and cost efficient and therefore a very useful tool not only for wineries but also for yeast suppling companies. In the current study the selection some yeast strains and their direct application in the wine industry is not possible since more analysis of oenological, biochemical and aromatic point of view should be implied.

Wine samples collected at the end of AF revealed the great predominance of *S. cerevisiae*, with a high intraspecific biodiversity (Garofalo et al., 2016). Furthermore, *S. cerevisiae* strains are mostly selected as starter cultures due to their unique biotechnological characteristics, such as fermentation capacity, the production of alcohol and CO_2_ and its resilience to adverse conditions of low pH and osmolality (Dimopoulou et al., 2020). The strain collection created in this research verifies the abovementioned observations. The identified different strains varied in richness and evenness among the wine samples, indicating the complex microbial interactions that occur during spontaneous fermentation. Microbial interactions play a dominant and complex role during AF. Despite the presence of multiple *S. cerevisiae* strains, some of which exhibited off-flavor characteristics during the final fermentation stage, none of the wine samples exhibited an off-flavor odors. It is well known that some indigenous strains persist in wineries for multiple years and are referred to as resident strains (Le Jeune et al., 2006). Particularly intriguing is the isolation of a single strain from samples from all regions, vintages and different varieties. This strain could be considered as a universal *S. cerevisiae* strain in Greek terroir. The genetic evaluation of this strain and its expansion all over Greece would be rather intriguing and further research is proposed. Martínez et al. (2004) propose a procedure that could be used as a tool for evaluating if a native isolate derives from the region where it was collected or if it is a strain derived from a commercial strain by microevolution. It is important to highlight that this strain showed different genomic fingerprint based on interdelta analysis among 5 different commercial strains and also it was isolated from a winery which has never used commercial strains. According to a previous study, two *S. cerevisiae* strains were isolated from the final stage of fermentation from different wineries of Beijing and possess important region-specific oenological characteristics (Sun et al., 2009). Finally, it is worth noting that 50% of the isolated strains were found in both white and red wines, suggesting that the assertion of certain strains being exclusively suitable for white or red vinification is questionable.

## Conclusion

The aim of this study was to develop a novel, rapid and applicable method for preliminary yeast preliminary selection of alcoholic fermentation starters in wine. The proposed phenotypic classification method was validated by the results of fermentation kinetics and sensory evaluation of the tested *S. cerevisiae* strains. A future perspective is to focus on some of the strains that are presented in the current survey, perform bigger volume laboratory fermentations, large scale pilot fermentations and afterwards to propose new functional indigenous yeast to the wine industry. Additionally, there is no doubt that it would be quite interesting in the future to test the proposed classification test in a larger collection. Interestingly, the geographical distribution of the species revealed the presence of one ubiquitous strain with great oenological potential. Further research work could be done for the evaluation of this unique strain under large scale fermentation in order to examine the commercialization potential by the wine industry.

## Data availability statement

The original contributions presented in the study are included in the article/Supplementary material, further inquiries can be directed to the corresponding author.

## Author contributions

AT: Conceptualization, Data curation, Formal analysis, Investigation, Methodology, Software, Writing – original draft, Writing – review & editing. VT: Data curation, Formal analysis, Methodology, Software, Writing – original draft. IP: Funding acquisition, Project administration, Resources, Supervision, Visualization, Writing – review & editing. MD: Conceptualization, Investigation, Methodology, Project administration, Supervision, Visualization, Writing – review & editing, Writing – original draft.
