# Supplementary material for: Developing a novel selection method for alcoholic fermentation starters by exploring wine yeast microbiota from Greece
Source: Front Microbiol. 2023 Dec 20;14:1301325. doi: 10.3389/fmicb.2023.1301325 (PMC10765506; doi:10.3389/fmicb.2023.1301325)
Supplement: Supplementary file 1 [file Data_Sheet_1.docx]

Supplementary Material

## Supplementary Table 1. List of commercial *S. cerevisiae* strains tested with interdelta fingerprint analysis

| **Strain** | **Company** |
| --- | --- |
| ZYMAFLORE® SPARK | Laffort, France |
| EXCELLENCE® FTH | Lamothe-Abiet, France |
| EnartisFerm ES181 | Enartis, Italy |
| Vin 13 | Anchor, France |
| EnartisFerm Red Fruit | Enartis, Italy |
| Vitilevure® 3001 Yseo | Martin Vialatte, France |
| SafŒno™ UCLM S325 | Fermentis, France |
| VIALATTE FERM® R82 | Martin Vialatte, France |
| EnartisFerm ES Floral | Enartis, Italy |

## Supplementary Figure 1.


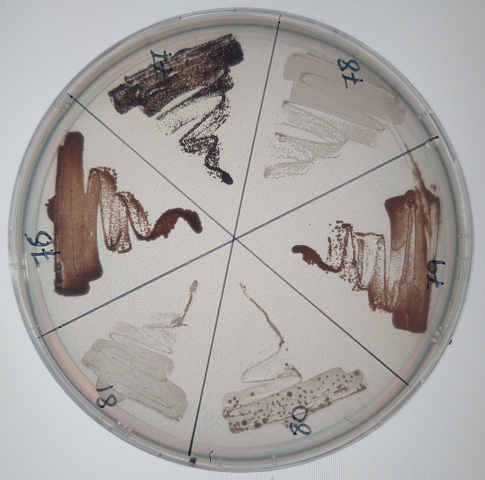

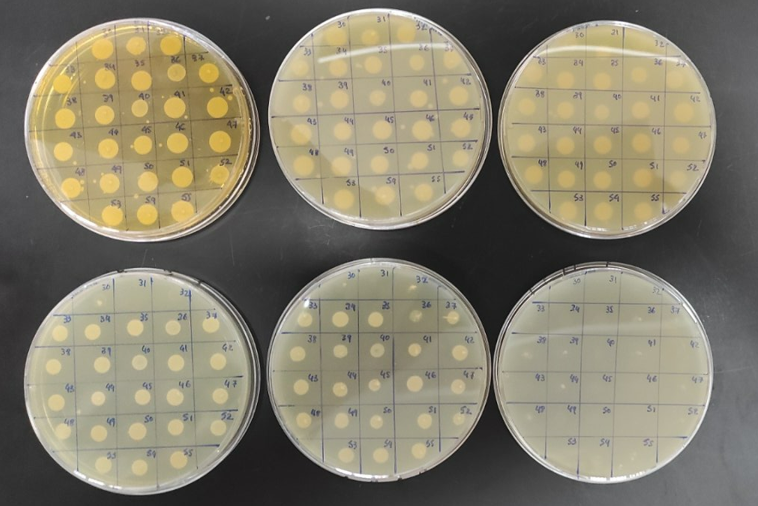

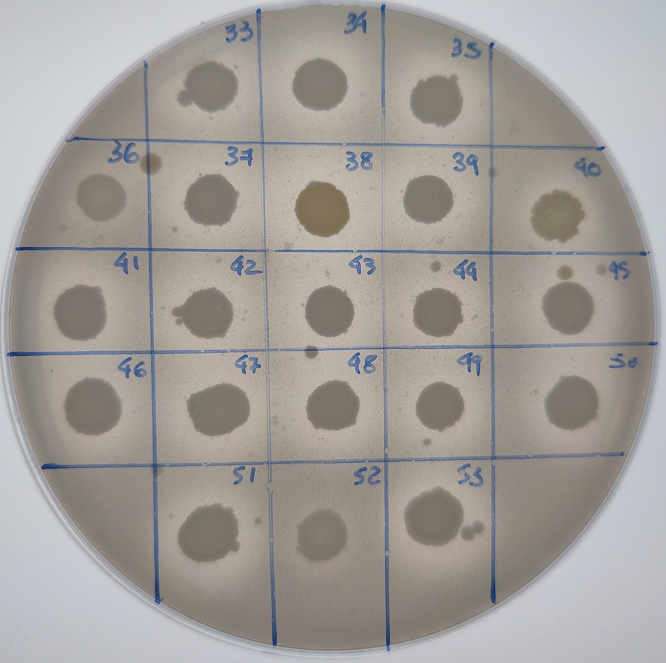

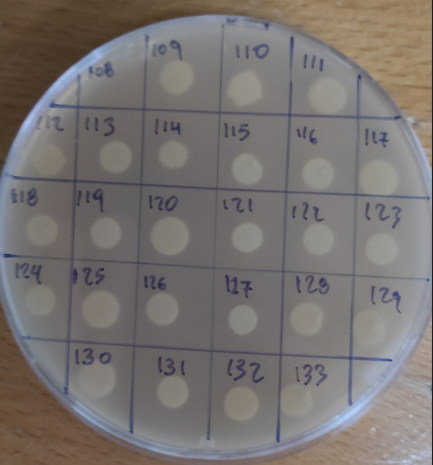

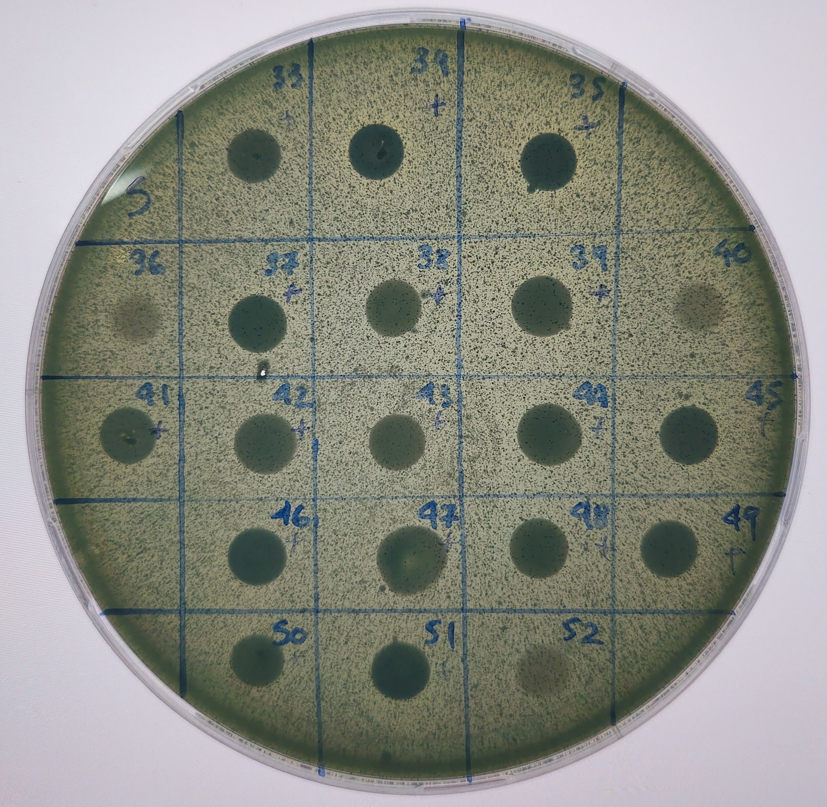


**(vi)**

**(v)**

**(iv)**

**(e)**

**(i)**

**(ii)**

**(iii)**

**(a)**

**(b)**

**(c)**

**(d)**

## Supplementary Figure 1. Incubated agar plates after incubation of the colonies in the different phenotypic tests: (a) killer toxin substrate, (b) CaCO_3_ substrate, (c) YNB- arbutin substrate, (d) Biggy agar and (e) YPD agar (pH =3) with i) 0 mg/L SO_2_, ii)100 mg/L SO_2_ iii)200 mg/L SO_2_ iv)300 mg/L SO_2_ v) 400 mg/L SO_2_ vi) 500 mg/L SO_2_.

## Supplementary Figure 2.


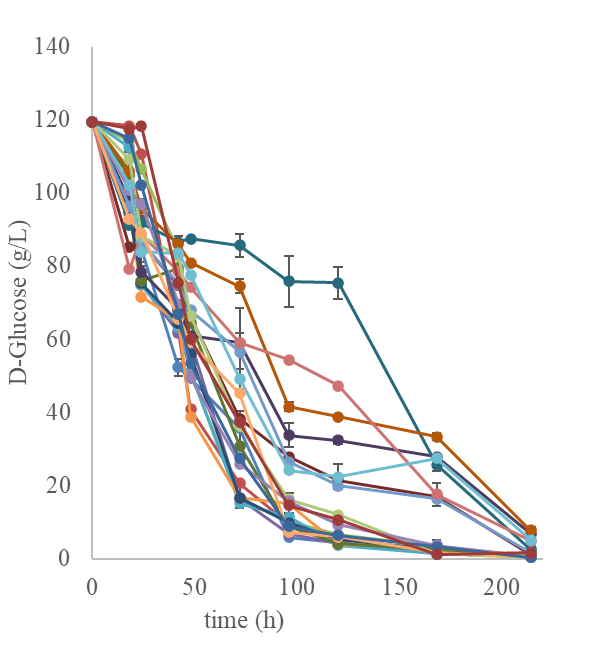

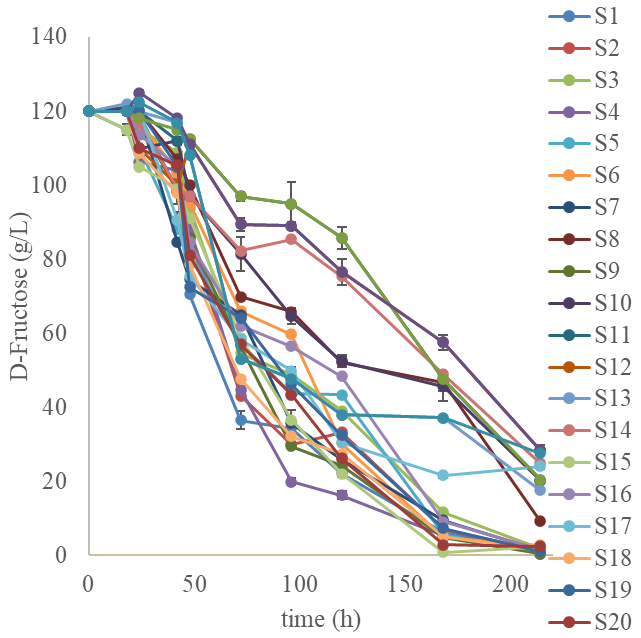


**Supplementary Figure 2.** Changes in sugar consumption [(a) D- glucose and (b) D- fructose] during alcoholic fermentations for the 20 *S. cerevisiae* strains
